# The Views of Hospital Secretaries

**Published:** 1911-06-03

**Authors:** 


### THE VIEWS OF HOSPITAL SECRETARIES.

**Mr. J. J. BARRON, Secretary-Superintendent, the Royal Infirmary, Bradford.**

I think the first result of the National Insurance Bill will be to decrease the number of our out-patients, though I do not believe there will be any difference so far as the number of in-patients is concerned. Then I fear it will have an unsettling effect on hospital finances for a year or two, as there will be a temptation on the part of employers and workpeople to withhold the contributions they have hitherto given for the upkeep of the medical charities. I believe this effect will be only temporary, as both of these classes must soon realise that in a large industrial centre like Bradford, a general hospital properly equipped and staffed, is not only an absolute necessity for the treatment of sick and injured workpeople, but also a great advantage to the employers.

Roughly, one-third (£4,066) of our annual income is received from the Bradford Hospital Fund (Incorporated), which has organised the collections in mills, workshops, and places of worship, and which raises also a large sum each year from galas and other outdoor functions. Our annual subscriptions and special maintenance contributions amount to £5,000, leaving a balance of £4,000 to be made up by dividends, interest, donations, and miscellaneous receipts. As our present income is barely sufficient for our needs, any diminution in our receipts would seriously affect the work of the Infirmary, and I am certain that, as soon as the public realise that sick and injured members of the working classes are having their chances of recovery prejudiced by the decrease in the hospital funds, there will be a return to the present methods of supporting the hospitals.

The weak part of the Bill is that no provision seems to be made for the treatment of serious surgical cases, and the claims of the hospitals appear to have been entirely forgotten. I trust that when the Bill is discussed some means will be devised to allocate to hospitals a considerable portion of the amount earmarked for medical benefit, and I think this can be done without Government interference with the control of these institutions. I cannot say what the attitude of the honorary medical staff will be on the question, but, if the provisions as to the amount to be paid to medical practitioners are dropped, I see no reason to fear that our medical staff will not in the future be as ready to do their share of service for the relief of sickness and suffering as they have been in the past.

The Bill has been announced at rather an inopportune time for our new Infirmary building scheme. The date for sending in designs by competing architects was June 1, and immediately thereafter the Assessor and the Building Scheme Committee proceeded to consider the plans submitted. When the adjudicators' decision has been given, I expect the successful plans will be exhibited, and the public of Bradford and the district will be informed that in the opinion of the Committee these plans show a hospital which Bradford ought to have, and the decision as to whether such a building will be erected must rest with the Bradford people. Promised subscriptions for the new scheme amount to nearly £80,000, and the Committee have deferred taking action to obtain additional contributions to this fund until they were in a position to present the accepted plans.

Our existing buildings are old and overcrowded, and quite incapable of accommodating the number of in-patients applying for treatment. We have a list of cases waiting for admission which averages about forty per week, clearly showing that a new and larger hospital is necessary to deal with all the patients who require medical or surgical aid. I do not anticipate that under the new conditions which the National Insurance Bill will bring into existence the number of hospital in-patients will be decreased, and the probability is that the need for hospitals will be greater than ever. It therefore rests with the well-wishers of hospitals to devise some method of conserving as far as possible the voluntary principle which has proved such a boon to our country and of formulating some scheme whereby the poorer members of the community may rely on obtaining that medical and surgical assistance which the hospitals with their splendid medical staffs have hitherto provided for those in need of it.

